# Validation of the Neuroception of Psychological Safety Scale (NPSS) Among Health and Social Care Workers in the UK

**DOI:** 10.3390/ijerph21121551

**Published:** 2024-11-23

**Authors:** Nicola Cogan, John Campbell, Liza Morton, David Young, Stephen Porges

**Affiliations:** 1 Department of Psychological Sciences and Health, University of Strathclyde, Glasgow G1 1QE, Scotland, UK; john.campbell.2022.100@uni.strath.ac.uk; 2 Psychology Department, Caledonian University, Glasgow G4 0BA, Scotland, UK; liza.morton@gcu.ac.uk; 3 Department of Mathematics and Statistics, University of Strathclyde, Glasgow G1 1XH, Scotland, UK; david.young@strath.ac.uk; 4 Traumatic Stress Research Consortium, Kinsey Institute, Indiana University, 150 S Woodlawn Avenue, Bloomington, IN 47405, USA; sporges@iu.edu

**Keywords:** psychological safety, polyvagal theory, psychometric validation, health and social care workers, mental health, trauma, post-traumatic growth, compassionate care, trauma-informed practice

## Abstract

Psychological safety is essential for rest, recovery, and fostering social connections, particularly for health and social care workers (HSCWs) who frequently operate in high-pressure environments. These workers are prone to traumatic stress, which can elevate their sense of threat and undermine their psychological safety. This study aimed to validate the Neuroception of Psychological Safety Scale (NPSS) among HSCWs in the UK (*n* = 443). The NPSS is based on polyvagal theory and assesses the dimensions of compassion, social engagement and bodily sensations. Internal consistency, test–retest reliability, convergent, discriminant, and concurrent validity were examined, along with the scale’s dimensionality. A three-factor structure was confirmed, with internal consistencies ranging from acceptable to excellent across subscales. Validity was supported by significant associations with measures of team psychological safety, well-being, post-traumatic stress, burnout, body perception, and personality. The NPSS also demonstrated strong test–retest reliability. These results validate the NPSS as a reliable and multidimensional tool for assessing psychological safety in health and social care settings. The study highlights the importance of psychological safety for HSCWs and provides a valuable measure to support interventions aimed at fostering safer and more supportive work environments.

## 1. Introduction

Psychological safety is essential for health and social care workers (HSCWs) due to its profound impact on both their well-being and the quality of patient care [1]. When workers feel psychologically safe, they are more likely to communicate openly, collaborate effectively, and seek support without fear of repercussions [2]. This environment not only reduces traumatic stress levels among workers but also enhances their overall mental health, enabling them to perform their roles more effectively [3]. Improved teamwork and communication fostered by psychological safety directly translate into better patient outcomes, as care is delivered more efficiently and with fewer errors [4]. Moreover, organisations that prioritise psychological safety tend to attract and retain talent more effectively, as workers are more inclined to stay in environments where they feel valued and supported [5]. This, in turn, reduces turnover rates and ensures continuity of care [6].

Psychological safety also encourages innovation and problem-solving within healthcare settings [7]. Workers who feel safe to voice ideas, take risks, and experiment with new approaches are more likely to contribute to advancements in patient care practices and operational efficiency [8]. Additionally, in contexts where ethical dilemmas are prevalent, psychological safety allows for open discussion and consideration of diverse viewpoints, ensuring that decisions are made with integrity and sensitivity to all stakeholders, including patients and their families [9]. Furthermore, psychological safety supports ongoing professional development by creating an environment where constructive feedback is valued and learning opportunities are embraced [10].

A well-established body of research focusing on the measurement and application of psychological safety within teams has demonstrated the importance of psychological safety within a range of organisational contexts [11,12,13,14,15]. However, psychological safety for HSCWs is crucial not only for organisational dynamics and patient care but also from the perspective of individual psychological safety. While several assessment instruments have been developed to evaluate constructs related to psychological safety and mental well-being, there remains a need for tools specifically tailored to assess psychological safety and its influence on both the individual and organisational outcomes of HSCWs. Instruments such as the Team Psychological Safety Scale (TPSS), Compassion Scale (CS), and Short Warwick-Edinburgh Mental Wellbeing Scale (SWEMWBS) have established validity and reliability [16,17,18]; however, they do not capture psychological safety as experienced at the individual level. A comprehensive review of the existing tools reveals the need for targeted assessment instruments that reflect the individual HSCWs’ perspective on psychological safety in high-stress working environments. This study presents the Neuroception of Psychological Safety Scale (NPSS) as a novel instrument specifically designed to address this gap, contributing valuable insights to the ongoing discourse on the importance of psychological safety in relation to the mental health and well-being of HSCWs.

Polyvagal theory (PVT) provides a comprehensive framework for understanding the vital role of psychological safety in health and social care settings, particularly for individuals. The theory is rooted in the neurophysiological processes of the vagus nerve, a major component of the parasympathetic nervous system that supports the body’s ability to rest, digest, and recover [19]. According to PVT, the autonomic nervous system operates through a hierarchical response system with three primary states: the ventral vagal state (associated with safety and social engagement), the sympathetic nervous state (linked to fight-or-flight responses), and the dorsal vagal state (related to immobilisation or shutdown in response to overwhelming threat). One of PVT’s key concepts is “neuroception”, which describes how the nervous system subconsciously scans the environment for signs of safety or danger. Unlike perception, which is largely conscious, neuroception occurs below conscious awareness, constantly assessing the surroundings to determine whether they are safe or threatening. This process activates corresponding physiological, emotional, and behavioural responses designed to either enhance social engagement when safe or trigger defensive responses when a threat is detected. This mechanism helps explain how people may react strongly to stressful or traumatic situations, even if they are not consciously aware of an immediate threat.

In healthcare environments, where stress and trauma are often prevalent, PVT highlights the importance of fostering psychological safety to support the well-being of both patients and healthcare professionals [20]. By ensuring a sense of safety, HSCWs are better able to regulate their emotions, connect with others, and perform effectively in high-pressure situations, ultimately enhancing overall care and recovery. PVT has helped to inform mental health, medical, and educational practices in the use of safe therapeutic presence, recognition of client’s non-verbal safety-signalling, interpreting representations of fear and safety in art therapy, investigating schema modes as means of coping, exploring the impact of multi-generational trauma through movement expressions, and processing physiological manifestations of trauma in military veterans [21,22,23,24,25,26]. PVT offers a comprehensive overview of safety from neurophysiological, psychological, and evolutionary theories [27,28]. When HSCWs feel psychologically safe, it corresponds to a state where their autonomic nervous system perceives minimal threat, allowing them to engage fully in social interactions and professional duties without being in a constant state of defence or hypervigilance [29]. In practical terms, a psychologically safe environment aligns with the PVT’s concept of the “social engagement system”, where individuals feel safe to connect, communicate, and collaborate effectively [30]. This mode also encourages pro-social behaviours, such as compassion. It activates the ventral vagal complex, which is linked to feelings of safety and connection, thereby enhancing emotional regulation, reducing stress, and promoting overall well-being among HSCWs [1].

Compassion is a cornerstone of health and social care delivery, and it is essential that HSCWs feel psychologically safe at work so they can offer compassion to patients [31]. As relational beings, we communicate psychological safety through compassion. Compassion has a positive effect on the physical, social, and psychological health of both the giver and receiver. Compassionate care helps patients to feel safe and increases engagement and participation in treatment or interventions [32]. It is linked with an increase in their hope for recovery, accountability, control over their health, and satisfaction. It also leads to the provision of safer care and the resilience of HSCWs [33]. Compassion is fundamental to relationship-based care and understanding of patients. A lack of compassion is a recognised threat to patient safety, and shortcomings have been reported in health systems due to a lack of compassion in health and social care delivery [34].

Social engagement is another critical aspect influenced by psychological safety [35]. In a psychologically safe environment, HSCWs are more likely to engage with their colleagues, patients, and the broader community. This engagement fosters a sense of belonging and mutual support, which can mitigate feelings of isolation and burnout [36]. Enhanced social interactions among HSCWs contribute to a more cohesive and collaborative team, ultimately benefiting patient care. Moreover, patients who perceive their caregivers as socially engaged are more likely to trust them and participate actively in their own care, which can lead to better health outcomes [37].

Body sensations, or the awareness of one’s own body and its sensations, also play a significant role in the well-being of HSCWs [38]. Psychological safety supports awareness of body sensations by creating an environment where workers can attend to their own physical and emotional needs without fear of judgement [39]. When workers are attuned to their own bodies, they are better able to manage stress, prevent burnout, and maintain overall health [40]. This self-awareness can also enhance empathy and the ability to provide compassionate care, as workers who are in tune with their own physical and emotional states are more likely to recognise and respond to the needs of their patients [41]. Conversely, environments lacking psychological safety may trigger defensive responses, such as activation of the sympathetic nervous system (fight-or-flight response) or even shutdown responses (dorsal vagal complex), which can hinder effective teamwork, communication, and decision-making among health and social care teams [42]. Therefore, integrating principles of PVT into health and social care settings can provide valuable insights into fostering psychological safety, enhancing interpersonal relationships, and ultimately improving both staff satisfaction and patient outcomes. By prioritising psychological safety through a polyvagal lens, health and social care organisations can create environments where workers thrive emotionally, professionally, and in their dedication to delivering compassionate and effective care [43]. This holistic approach, which includes compassion, social engagement, and body sensations, ensures that both workers and patients thrive in a supportive and nurturing environment [44].

Considering the relevance of psychological safety in preventing, mitigating, and treating trauma-related conditions, we previously reported on the development of the NPSS [45]. The NPSS, grounded in PVT, is the first psychometric tool that aims to measure psychological safety for the individual. This scale incorporates three key factors—compassion, social engagement, and bodily sensation—which are integral to understanding the neurobiological foundations of psychological safety. Compassion reflects the ability to engage with others in a supportive and non-judgemental manner, promoting trust and connection. Social engagement refers to the capacity for meaningful social interactions, which are essential for fostering a sense of safety and belonging. Bodily sensation, linked to the body’s physiological responses, highlights the importance of autonomic regulation and bodily awareness in feeling safe and grounded. These dimensions collectively support the prevention, mitigation, and treatment of trauma-related conditions by facilitating an environment where the autonomic nervous system can shift into a state of safety and regulation. The three-factor structure of the NPSS showed adequate fit, and good reliability. The scale has been widely adopted across a range of settings including evaluation of Eye Movement Desensitisation and Reprocessing (EMDR) in dissociative disorders; exploring the reintegration of children and youth experiencing homelessness and instability; examining the significance of feeling safe for resilience of adolescents in sub-Saharan Africa; and as an informative measure for a model of Human-Animal Interactions [46,47,48,49]. In addition, researchers have validated an Italian version of the scale in a non-clinical sample and the scale was found to have a three-factor structure with good convergent, divergent and test–retest validity and robust psychometric properties [50,51]. Our current work aimed to further develop and strengthen the psychometric properties of the NPSS with a sample of HSCWs, testing the scales’ convergent, discriminant, and concurrent validity, as well as its dimensionality. To contextualise the development and application of the NPSS, recent studies using similar instruments to measure factors related to psychological safety, such as compassion, social engagement and bodily sensations, were reviewed. Instruments such as the TPSS and the CS have been widely applied in health and social care settings [16,17], demonstrating the importance of measuring these dimensions to enhance well-being and reduce stress among HSCWs. These studies provide a foundation for understanding the value of psychological safety metrics and highlight the relevance of the NPSS in addressing similar constructs within health and social care environments.

## 2. Materials and Methods

Ethical approval for this research was sought and granted from the University Ethics Committee (33/02/12/2020/A) and all participants provided informed consent to their participation prior to engaging with the research. Participants were recruited through convenience sampling via social media platforms such as Instagram, Facebook, Twitter, and LinkedIn, and the University’s online research participant recruitment system. Participants were selected based on specific inclusion and exclusion criteria: eligible participants were adults (18 years or older) residing in the UK and worked in health or social care roles. Exclusion criteria included conditions potentially impacting comprehension, such as dementia, current substance dependence, or severe neuropsychiatric disorders requiring hospitalisation. To ensure comprehension and accuracy, participants were provided with a participant information sheet that included a preliminary review of a sample of the NPSS questions and validation scales, with opportunities to clarify instructions before proceeding. The survey was administered electronically through a secure online platform (Qualtrics) [52], providing consistency in format and accessibility. Further recruitment efforts included outreach to third-sector charities and health and social care organisations to reach eligible participants. Despite these efforts, the sample exhibited some demographic variation that may not fully represent the broader health and social care workforce. Midway through data collection, monitoring indicated a low response rate from males and adults from ethnic minority groups. To address underrepresentation, targeted recruitment efforts were made through data collection, specifically aimed at increasing diversity and inclusion of participants who were under-represented. These adjustments sought to improve sample heterogeneity and better capture the perspectives of a wider range of individuals within the health and social care field, although limitations in representativeness remain.

In completing the online survey, participants first provided demographic information, including gender, age, ethnicity, country of residence, employment status, and health status. They then completed the 29-item NPSS [45], followed by seven psychometrically validated measures. Upon completing the survey, participants received a debriefing, which included information on relevant support agencies and the contact details of the lead researcher for any follow-up questions about the study. Data collection took place from November 2023 to May 2024.

The online survey included the NPSS along with several measures related to psychological safety: the Team Psychological Safety Scale (TPSS), the Compassion Scale (CS), the Body Perception Questionnaire-Very Short Form (BPQ-VSF), the Short Warwick-Edinburgh Mental Wellbeing Scale (SWEMWBS), the Burnout Measure-Short Version (BMS), and the Abbreviated Post-Traumatic Stress Disorder Checklist-Civilian (APCL-C) [16,17,45,53,54,55]. Additionally, we included a measure that we hypothesised would not be strongly related to psychological safety: the Ten-Item Personality Inventory (TIPI) [56]. (See Table 1 for a full description of the measures).

Statistical analyses were conducted using the Statistical Package for the Social Sciences (SPSS 28) to calculate descriptive statistics, assess internal reliability and conduct preliminary analyses [57]. Confirmatory Factor Analysis (CFA) was performed using R to evaluate the structural validity of the NPSS [58]. Model fit indices, including the Comparative Fit Index (CFI), Tucker–Lewis Index (TLI), and Root Mean Square Error of Approximation (RMSEA), were calculated to confirm the three-factor model’s adequacy. These combined tools provided robust insights into the scale’s psychometric properties, to test both its reliability and validity across various dimensions.

To maximise the use of available data and minimise biased estimates, missing item data were handled using an imputation method. Given that the data appeared to be missing at random and the missing rate was low (1.17%), a single imputation method was deemed appropriate [59]. Descriptive statistics, including means, standard deviations, minimum and maximum values, and percentages for categorical data, were used to characterise the sample and assess the psychometric properties. These statistics also provided insights into normality and the presence of potential outliers. The internal reliability of the NPSS was evaluated using Cronbach’s α. Test-retest reliability over a three-week interval was assessed using intra-class correlation coefficients, with data obtained from a sub-sample (10%) of the total sample. Convergent validity was examined by evaluating the correlations between the NPSS and other relevant measures (see Table 1), including the TPSS, CS, BPQ-VSF, SWEMWBS, BMS, and APCL-C [16,17,45,53,54,55]. Discriminant validity was assessed by examining the correlations between the NPSS and the TIPI [56]. The measures included in this study have been widely used in research exploring mental health and well-being among HSCWs and are validated instruments that are recognised for their reliability and relevance in assessing constructs crucial to understanding psychological safety. Moreover, these scales formed the basis of earlier work in developing the NPSS, where they contributed significantly to establishing the scale’s foundational structure and content validity [45].

**Table 1:** Validation measures.

Measure	Description	Rating/Scoring	Psychometrics
Neuroception of Psychological Safety Scale (NPSS) [45]	The NPSS is a 29-item self-report measure with three sub-scales: social engagement, compassion, and body sensations.	Participants rate how well statements describe their feelings over the past week, rating statements such as “I felt valued”, “I felt compassion towards others”, and “My heart rate felt steady”. Responses are on a 5-point Likert scale from 1 (strongly disagree) to 5 (strongly agree). Higher NPSS scores indicate higher levels of individual psychological safety.	The NPSS showed good internal reliability with Cronbach’s α of 0.95 overall, and sub-scale α of 0.94 for social engagement, 0.90 for compassion, and 0.93 for bodily sensations [60].
Team Psychological Safety Scale (TPSS) [16]	The TPSS is a 7-item scale with both positively and negatively worded statements measuring psychological safety in a team context.	Participants rate statements like “If you make a mistake on this team, it is often held against you” and “It is safe to take a risk on this team” on a 7-point Likert scale from 1 (very inaccurate) to 7 (very accurate). Higher TPSS scores indicate a safer team environment.	The TPSS has good internal consistency and reliability [16,61].
Compassion Scale (CS) [17]	The CS is a 16-item scale assessing compassion through constructs of kindness, common humanity, mindfulness, and reduced indifference.	Participants rate statements like “I like to be there for others in times of difficulty” and “I am unconcerned with other people’s problems” on a 5-point Likert scale from 1 (almost never) to 5 (almost always). Higher scores indicate greater compassion for others.	The CS has demonstrated reliability and validity [62].
Body Perception Questionnaire-Very Short Form (BPQ-VSF) [53]	The BPQ-VSF is a 12-item measure assessing subjective bodily experiences related to autonomic nervous system functions.	Participants rate their awareness of characteristics such as “How fast I am breathing” and “Stomach and gut pains” on a 5-point Likert scale from 1 (never) to 5 (always). Higher scores suggest a dysregulated autonomic nervous system and poorer health outcomes.	The BPQ-VSF is reliable and valid [63].
Short Warwick–Edinburgh Mental Wellbeing Scale (SWEMWBS) [18]	The SWEMWBS is a 7-item scale which briefly assesses mental wellbeing.	Participants reflect on their thoughts and feelings over the past two weeks and rate statements like “I’ve been feeling useful” and “I’ve been feeling close to other people on a Likert scale from 1 (none of the time) to 5 (all of the time). Higher scores indicate higher mental wellbeing.	The SWEMWBS has good internal consistency and reliability [64,65].
Burnout Measure-Short Version (BMS) [54]	The BMS is a 10-item short measure to assess burnout across various contexts.	Participants rate how often they feel “Tired”, “Worthless/Like a failure”, and “I’ve had it” on a 7-point scale from 1 (never) to 7 (always). Higher scores indicate higher levels of burnout.	The BMS has good psychometric properties [66].
Abbreviated Post-Traumatic Stress Disorder Checklist-Civilian (APCL-C) [55]	The APCL-C is a 6-item measure assessing symptoms of post-traumatic stress in civilians.	Participants rate how often they have been bothered by symptoms such as “Repeated, disturbing memories” and “Feeling irritable or having angry outbursts” on a 5-point Likert scale from 1 (not at all) to 5 (extremely). Higher scores indicate potential post-traumatic stress requiring further attention.	The APCL-C has adequate internal consistency and reliability [67].
Ten-Item Personality Inventory (TIPI) [56]	The TIPI measures the Big-5 personality domains: extraversion, agreeableness, conscientiousness, emotional stability, and openness to experience.	Participants rate pairs of traits on a 7-point Likert scale from 1 (disagree strongly) to 7 (agree strongly). Each domain is represented by two statements, one positive and one negative, requiring reverse scoring for half the items. Higher scores indicate strong identification with the associated personality traits.	The TIPI is valid and reliable [68].

Concurrent validity was determined through logistic regressions to assess whether the NPSS could predict trauma exposure (dependent variable: trauma yes/no). Sum scores of the NPSS were calculated and used in validity testing. Known groups’ validity was explored using an independent *t*-test to compare NPSS scores by gender and the impact of occupational trauma [69,70]. All statistical tests were two-tailed, with *p*-values of less than 0.05 considered statistically significant. The study process and materials are outlined in Table 2.

## 3. Results

The analysis included 443 complete responses from HSCWs. Participants were most likely to be employed in a nursing role, followed by medical doctor and applied health professionals; with the majority employed full-time. Length of service ranged from 6 months to 25 > years (*M* = 8.8; *SD* = 7.9) and level of seniority was predominantly disclosed as “Intermediate” (42.7%). Participants’ ages ranged from 18 to 68 years (*M* = 38.5, *SD* = 11.8). The majority identified as White-British (68.7%), mostly residing in England (48.5%). Additionally, 23.9% of participants reported long-term physical health issues, and 31.2% disclosed diagnosed mental health issues. For a detailed summary of participants’ socio-demographic characteristics, see Table 3.

### 3.1. Internal Reliability

Descriptive statistics for NPSS sum scores and relevant validation measures are presented in Table 4. Although NPSS scores deviated from normality, as indicated by skewness, kurtosis, and Shapiro-Wilk tests (all *p* < 0.05), research suggests that Cronbach’s α is generally robust even in non-normally distributed data, especially with large sample sizes [71]. The NPSS data showed a mean sum score of 109.41 (*SD* = 16.09) and an item mean of 3.77, with slight negative skewness (−0.21) and moderate positive kurtosis (0.50). Despite these deviations, the internal consistency of the NPSS was strong, with an overall Cronbach’s α of 0.946, indicating excellent reliability. Subscale analyses also demonstrated high internal reliability, with α values of 0.896 for compassion, 0.937 for social engagement and 0.930 for bodily sensations, reflecting good to excellent reliability [72]. Notably, the exclusion of any items did not improve α values, suggesting that all items consistently contribute to the scale’s reliability. This strong internal reliability indicates that the NPSS is a reliable measure of psychological safety, even with deviations from normality. Outliers, which can distort linear relationships and affect the accuracy of correlation and regression analyses, were removed to ensure a more accurate representation of the relationships between variables [73].

### 3.2. Test–Retest Reliability

Test–retest reliability at three weeks in the sub-sample was (*r* = 0.87; *p* < 0.01), indicating high reliability for the NPSS. This suggests that the NPSS demonstrates consistent results over time within this sample.

### 3.3. Convergent and Discriminant Validity

We evaluated convergent validity by correlating the NPSS with several related constructs, including TPSS, CS, BPQ-VSF, SWEMWBS, BMS, and APCL-C. The significant correlations between NPSS scores and these measures provide strong evidence of convergent validity, confirming that NPSS effectively captures elements associated with psychological safety. To assess discriminant validity, we examined the correlations between NPSS mean scores and the TIPI. As expected, these correlations were positive but generally weaker than those observed with the other constructs (with the exception of the correlation between NPSS and the BPQ-VSF which was weak to moderate), indicating that while NPSS shares some variance with personality traits, it remains distinct. These findings collectively demonstrate that NPSS is a robust measure that reliably differentiates psychological safety from related but distinct constructs (see Table 5).

### 3.4. Concurrent Validity

Concurrent validity was evaluated using logistic regression to determine if the NPSS could accurately predict self-reported trauma exposure (yes/no). The logistic regression model was statistically significant, *X*^2^(22) = 207.63, *p* < 0.001. It explained 17.1% (Nagelkerke R^2^) of the variance in trauma exposure and correctly classified 78.3% of cases. The NPSS successfully distinguished between participants who reported trauma exposure and those who did not, demonstrating good concurrent validity.

### 3.5. Post Hoc Analysis

An independent samples *t*-test revealed no significant difference in NPSS sum scores between males (*M* = 109.82, *SD* = 17.19) and females (*M* = 109.30, *SD* = 15.60), *t*(212.77) = 0.29), (*p* = 0.385), with a small effect size (Cohen’s *d* = 0.03). This indicates that gender does not play a significant role in influencing NPSS scores among HSCWs. Conversely, a separate independent samples *t*-test showed a significant difference in NPSS sum scores between participants who reported that exposure to occupational trauma had negatively impacted their thought processes and those who did not. Participants who answered “Yes” to the trauma impact question had significantly lower NPSS scores (*N* = 184; *M* = 105.36; SD = 15.88) compared to those who answered “No” (N = 193; M = 114.71; *SD* = 14.84), (*t*(370.09) = −5.90), (*p* < 0.001), with a large effect size (Cohen’s *d* = 0.61). These findings suggest that exposure to occupational trauma is associated with a significant decrease in perceived psychological safety, as participants who reported negative impacts from trauma had lower NPSS scores. This aligns with theoretical expectations, highlighting the adverse effects of trauma on psychological well-being in the workplace.

### 3.6. Dimensionality

To examine the dimensionality of the NPSS, the original hypothesised 3-factor model (compassion, social engagement and body sensations) was compared with several logically derived alternative factor solutions based on interdimensional correlations. This approach is commonly used in studies assessing dimensionality [74,75]. The original 3-factor model demonstrated the best fit to the data, with all 29 items loading appropriately on their respective factors. Goodness-of-fit indices demonstrated the model’s adequacy including CFI (0.87), TLI (0.85), and RMSEA (0.08). Generally, CFI and TLI values of 0.90 or above and RMSEA values below 0.08 are considered indicative of adequate model fit. Although the NPSS model achieved reasonable fit thresholds, these values suggest room for further refinement to enhance the model’s dimensionality and overall fit. Factor loadings (see Table 6) showed acceptable fit of items to the three factors and verified the structural validity of the NPSS [76].

## 4. Discussion

This study provides robust evidence validating the NPSS within a sample of UK HSCWs. Our findings reveal that the NPSS captures the nuanced dimensions of psychological safety, namely, compassion, social engagement and bodily sensations, which are central to an individuals’ experiences of psychological safety in a high-stress environment. The NPSS exhibited strong internal consistency, test–retest reliability, and favourable levels of convergent and discriminant validity, demonstrating its psychometric strength. Importantly, the NPSS also demonstrated concurrent validity by independently predicting trauma exposure, thus underscoring its potential utility in identifying individuals at higher risk within occupational settings. This study builds upon our previous work introducing the NPSS, grounded in PVT and makes a significant contribution to the growing field of operationalising and measuring psychological safety at the individual level [45]. To our knowledge, this research represents the first comprehensive validation of the NPSS among HSCWs in the UK, establishing its reliability, validity, and dimensionality within this specific population.

As the NPSS is a newly developed measure, studies validating its use across different populations are only beginning to emerge. The psychometric soundness of the NPSS was corroborated by studies conducted in different populations, including a UK community sample and an Italian version of the NPSS [50,51,60]. The current study and these recent validation studies of the NPSS support a statistically sound factor model that effectively delineates the core dimensions of psychological safety: “compassion,” “social engagement,” and “bodily sensations.” These dimensions align with key processes influencing an individual’s overall sense of psychological safety, including the calming effects of compassion, social connectedness, and bodily comfort [77,78]. The NPSS emerges as a novel, theoretically informed tool for assessing psychological safety, particularly valuable in clinical settings involving trauma survivors and those exposed to occupational trauma [44,79]. It provides clinicians, therapists, and researchers with a quantitative means to evaluate self-reported psychological safety, thereby informing therapeutic decisions tailored to individual tolerance zones; critical for effective trauma and psychological safety interventions [80,81]. Moreover, the implications of our study extend beyond clinical practice, encompassing broader applications in organisational culture and policy within health and social care organisations [82]. By accurately measuring psychological safety, the NPSS facilitates the identification of areas requiring intervention and the evaluation of initiatives aimed at enhancing workplace well-being and resilience. This capability is pivotal in supporting the mental health and performance of HSCWs, thereby contributing to overall improvements in service quality and patient outcomes.

A major strength of this study lies in its rigorous evaluation of the NPSS across critical psychometric dimensions, including reliability, validity, and dimensionality. Notably, the BPQ-VSF demonstrated a weak negative correlation with the total score on the NPSS. Upon further reflection, it seems that rather than serving as a validating scale, the BPQ-VSF may actually be capturing a different dimension that is more specific to body sensations and autonomic reactivity. When analysing the correlations between the NPSS subscales, we observed that the BPQ-VSF total score was weakly correlated with the NPSS subscales of compassion and social engagement, but moderately correlated with body sensations. These findings suggest that while the BPQ-VSF is related to certain dimensions of psychological safety, particularly those involving body sensations and autonomic reactivity, it may not be a comprehensive validating measure for psychological safety as a whole. Instead, it appears to tap into specific facets of psychological safety that are more closely related to body sensations and autonomic awareness than to processes of the mind involving cognitive, affective, and behavioural responses.

Given these nuances, resilience-focused treatment interventions designed to address autonomic nervous system dysregulation have been found to help mitigate risks to mental health and improve well-being in the workplace [83]. Future work could explore the impact of such interventions on HSCWs’ sense of psychological safety, particularly in environments characterised by high levels of stress and trauma exposure. Furthermore, incorporating trauma-informed care principles within such interventions may bolster their effectiveness, helping workers process and integrate their experiences more constructively. Understanding these dynamics could also inform targeted strategies to enhance both individual and team psychological safety in high-stress work settings.

Recognising the need for a more time-efficient tool, we are currently undertaking research to develop a shorter version of the NPSS, specifically designed for quick screening purposes. This condensed form aims to retain the most predictive and psychometrically robust items from the full scale while ensuring it still effectively captures the key dimensions of compassion, social engagement and bodily sensations. Our ongoing efforts involve using item response theory and factor analysis to identify the items that contribute most significantly to the scale’s overall reliability and validity. Once developed, this shorter version will undergo rigorous testing to confirm that it maintains the psychometric strengths of the full NPSS while offering the convenience of a brief, efficient screening tool.

Given the unique stressors and complexities in health and social care settings, further validation studies are necessary to explore the NPSS’s applicability with diverse groups and in varied occupational environments. This emerging research highlights the need for future studies to assess the NPSS’s effectiveness across broader demographic and professional samples, ensuring that the instrument accurately reflects psychological safety across diverse populations. These efforts will help refine the NPSS, enhancing its utility as a comprehensive measure of psychological safety in health and social care settings and beyond. Incorporating digital health tools and virtual reality environments, for example, could provide new avenues for testing psychological safety in innovative ways, potentially reaching broader audiences and enhancing intervention delivery. Additionally, cross-cultural validations could ensure the NPSS retains its relevance and applicability across different healthcare systems and sociocultural contexts. By integrating these findings, our future work aims to contribute to the development of safer, more supportive environments in health and social care, ultimately promoting the well-being of workers and the delivery of compassionate, relationally-based, trauma-informed patient care. Enhancing psychological safety through evidence-based, targeted interventions could lead to improved job satisfaction, reduced burnout, and better overall mental health and wellbeing outcomes for HSCWs.

However, despite the promising results of the current study, several challenges remain.First, while the NPSS has demonstrated strong psychometric properties, the goodness-of-fit indices were not optimal, suggesting room for improvement in the scale’s dimensionality. Future research could focus on refining the scale’s structure to achieve a better fit, ensuring it more accurately reflects the experiences of psychological safety among diverse populations. Second, this study relied on self-reported trauma exposure as an outcome variable, which may not fully capture the chronic adversities linked to autonomic nervous system functioning as emphasised in PVT. Expanding future studies to include more comprehensive measures of trauma and adversity would enhance the NPSS’s applicability in trauma-informed care. Additionally, the BPQ-VSF, used to measure body sensations, demonstrated only weak to moderate correlations with the NPSS’s dimensions. This raises questions about its suitability as a validating measure, suggesting that further exploration of alternative scales might yield stronger evidence for the NPSS’s validity. Finally, the applicability of the NPSS to different occupational environments and diverse demographic groups remains an area that requires further investigation.

Further research should also explore the application of the NPSS in emerging technologies, such as digital health platforms, artificial intelligence (AI)-driven mental health interventions, and real-time monitoring tools [84]. These technologies hold significant potential for enhancing the assessment and ongoing monitoring of psychological safety, particularly in high-stress environments like health and social care. Utilising AI could also enable more personalised and timely interventions, thereby improving outcomes for individuals at risk. Moreover, it is crucial to validate the NPSS with both children and young people and older adult populations, as their experiences of psychological safety may differ significantly from those of adults. Understanding how these dimensions manifest in such populations is essential for developing age-appropriate interventions and support mechanisms [85]. Validation of the NPSS in these groups could provide valuable insights into how psychological safety evolves across the lifespan and inform targeted strategies for fostering resilience and well-being throughout the life trajectory.

## 5. Conclusions

In conclusion, this research sought to validate the NPSS among HSCWs in the UK, examining its reliability, validity, and applicability in high-stress occupational settings. Our findings confirm the NPSS as a robust tool, demonstrating excellent internal consistency, test–retest reliability, and construct validity, supporting its use in measuring psychological safety within this population. The three-factor model comprising compassion, social engagement and bodily sensations highlights the critical roles these dimensions play in fostering psychological safety, well-being, and effective functioning among HSCWs. As hypothesised, the NPSS captures these key dimensions, making it a valuable tool for assessing psychological safety and informing targeted interventions. However, as this is an emerging measure, further validation studies with diverse demographic and occupational groups are recommended to ensure its broad applicability and sensitivity to unique stressors in various occupational settings and diverse sociocultural contexts. Looking ahead, the NPSS holds significant potential to advance multidisciplinary research, inform policy development, and support interventions targeting psychological safety in health and social care settings. We anticipate that the NPSS will serve as a valuable resource for policymakers, practitioners, and service users alike, offering a comprehensive tool to assess and discuss psychological safety in a variety of contexts. By enabling the measurement of psychological safety at the individual level, the NPSS will facilitate a deeper understanding of how safety is experienced within work environments, particularly in health and social care settings. This will allow for ongoing monitoring of changes over time, providing key insights into the effectiveness of interventions and policies. Ultimately, the NPSS has the potential to contribute to the creation of safer, more supportive environments that foster well-being, reduce trauma, and enhance both individual and organisational outcomes.

## Figures and Tables

**Table 2:** Study process and materials.

Stage	Description
1. Participant Pool	The initial pool consisted of HSCWs recruited based on inclusion criteria to ensure representativeness. Targeted outreach was employed to include diverse demographics, enhancing the study’s generalisability.
2. Recruitment and Consent	Participants were recruited through social media and university platforms. Consent was obtained electronically, ensuring all participants were informed of their rights, study aims, and data handling practices. Exclusion criteria were applied to ensure appropriate comprehension of survey questions.
3. Data Collection	Data were collected online using Qualtrics ensuring consistency and accessibility. Collection occurred over six months, with additional recruitment efforts made to encourage participation from under-represented groups to improve diversity.
4. Measurement Tools	The NPSS and other validated scales were used to assess dimensions such as team psychological safety, compassion, and well-being. Each scale was selected based on its relevance and established psychometric properties for reliability and validity within health contexts.
5. Data Analysis Process	Data were analysed using SPSS and R, applying methods such as Cronbach’s alpha for internal reliability, test–retest reliability, and factor analysis to confirm structural validity. Missing data were imputed where necessary, and descriptive statistics were calculated to characterise the sample.
6. Reliability and Validity Tests	Measures of internal consistency, test–retest reliability, convergent and discriminant validity were applied to assess the psychometric robustness of the NPSS. This involved examining correlations with related constructs and assessing fit indices through CFA.
7. Results Interpretation	Findings were interpreted within the context of existing literature, evaluating the extent to which the NPSS captured the intended dimensions of psychological safety for HSCWs. Significant associations with constructs such as trauma exposure were noted, highlighting NPSS’s potential use in identifying at-risk individuals.
8. Conclusions	Based on the results, the study validated the NPSS as a reliable tool for assessing psychological safety in health and social care settings. The study recommended NPSS for use in initiatives aimed at enhancing workplace well-being and psychological safety and suggested further research for its application in diverse settings. A shorter version of the NPSS was proposed for quicker screening.

**Table 3:** HSCWs socio-demographic data.

Variables	*N* (443)
Age	18–68 (*M* = 38.5; *SD* = 11.8)
Gender	
Female	311 (70.2%)
Male	126 (28.4%)
Non-binary	6 (1.4%)
Other	1 (0.2%)
Ethnicity	
White-British	304 (68.7%)
White-Other	30 (6.8%)
Asian	44 (10%)
Black	48 (10.9%)
Mixed/Multiple ethnicities	8 (1.8%)
Other	9 (2%)
UK residence	
England	215 (48.5%)
Scotland	175 (39.5%)
Wales	31 (7%)
Northern Ireland	12 (2.7%)
Health disclosure	
Diagnosed mental health issues	138 (31.2%)
Long-term physical health issues	106 (23.9%)
None of the above	240 (54.2%)
Other	24 (5.4%)
Prefer not to answer	10 (2.3%)

% calculations exclude missing data.

**Table 4:** Descriptive statistics for the NPSS and validation measures.

Measure	Mean	Median	SD	Skew	Kurtosis
NPSS	109.41	110	16.09	−0.214	0.503
TPSS	33.74	34	7.56	−0.474	0.222
CS	64.99	66	7.62	−0.546	0.242
BPQ-VSF	28.04	27	10.03	0.753	0.421
SWEMWS	24.27	24	4.82	−0.053	−0.016
BMS	108.12	114	29.82	−0.933	0.470
APCL-C	14.15	13	5.79	0.542	−0.452
TIPI extraversion	7.71	8	3.12	0.109	−0.695
TIPI agreeableness	10.51	10	2.08	−0.168	−0.651
TIPI conscientiousness	10.41	11	2.36	−0.458	−0.344
TIPI emotional stability	6.80	7	2.81	0.197	−0.548
TIPI openness to experience	9.72	10	2.39	−0.216	−0.453

**Table 5:** Correlation matrix of associations between the NPSS scores and measures used to test convergent and discriminant validity.

Measures	1	2	3	4	5	6	7	8	9	10	11	12	13	14	15
1. NPSS	-	0.917 **	0.696 **	0.779 **	0.480 **	0.376 **	−0.289 **	0.686 **	−0.621 **	−0.500 **	0.151 **	0.293 **	0.306 **	−0.337 **	0.109 **
2. NPSS social engagement			0.550 **	0.542 **	0.494 **	0.281 **	−0.213 **	0.608 **	−0.548 **	−0.420 **	0.167 **	0.251 **	0.202 **	−0.198 **	0.070
3. NPSS compassion				0.329 **	0.288 **	0.607 **	−0.050 *	0.364 **	−0.252 **	−0.142 **	0.139 **	0.356 **	0.204 **	−0.110 *	0.223 **
4. NPSS body sensations					0.326 **	0.154 **	−0.402 **	0.619 **	−0.636 **	−0.582 **	0.070	0.154 **	0.350 **	−0.485 **	0.044
5. TPSS						0.250 **	−0.177 **	0.364 **	−0.371 **	−0.354 **	0.083	0.254 **	0.190 **	−0.116 *	−0.050
6. CS							0.107	0.213 **	−0.087 **	−0.038	0.138 **	0.454 **	0.234 **	−0.075	0.280 **
7. BPQ-VSF								−0.282 **	0.452 **	0.501 **	−0.049	−0.023	−0.151 **	0.277 **	0.090
8. SWEMWS									−0.697 **	−0.539 **	0.207 **	0.223 **	0.363 **	−0.486 **	0.106 *
9. BMS										0.694 **	−0.129 **	−0.205 **	−0.341 **	0.525 **	−0.096 *
10. APCL-C											−0.068	−0.164 **	−0.230	0.517 **	0.010
11. TIPI extraversion												0.023	0.165 **	−0.146 **	0.251 **
12. TIPI agreeableness													0.344 **	−0.284 **	0.330 **
13. TIPI conscientiousness														−0.338 **	0.205 **
14. TIPI emotional stability															−0.196 **
15. TIPI openness to experience															-

** *p* < 0.01; * *p* < 0.05.

**Table 6:** Component loadings of the 3-factor solution of NPSS.

		Loading	
Statement Items (*N* = 29)	Factor 1	Factor 2	Factor 3
1. I felt valued	0.774		
2. I felt comfortable expressing myself	0.593		
3. I felt accepted by others	0.679		
4. I felt understood	0.796		
5. I felt like others got me	0.748		
6. I felt respected	0.783		
7. There was someone who made me feel safe	0.621		
8. There was someone that I could trust	0.648		
9. I felt comforted by others	0.750		
10. I felt heard by others	0.804		
11. I felt like people would try their best to help me	0.842		
12. I felt cared for	0.893		
13. I felt wanted	0.739		
14. I did not feel judged by others	0.565		
15. I felt able to empathise with other people			0.744
16. I felt able to comfort another person if needed			0.823
17. I felt compassion for others			0.854
18. I wanted to help others relax			0.789
19. I felt like I could comfort a loved one			0.683
20. I felt so connected to others I wanted to help them			0.730
21. I felt caring			0.748
22. My heart rate felt steady		0.870	
23. Breathing felt effortless		0.876	
24. My voice felt normal		0.762	
25. My body felt relaxed		0.782	
26. My stomach felt settled		0.811	
27. My breathing was steady		0.906	
28. I felt able to stay still		0.701	
29. My face felt relaxed		0.794	

## Data Availability

Anonymised data available on request.
